# Obstetrical health care inequities in a universally insured health care system

**DOI:** 10.1016/j.xagr.2023.100256

**Published:** 2023-07-23

**Authors:** Shara Fuller, Molly Kuenstler, Marie Snipes, Michael Miller, Monica A. Lutgendorf

**Affiliations:** 1 Department of Gynecologic Surgery and Obstetrics, Naval Medical Center San Diego, San Diego, CA (Drs Fuller and Miller); 2 Department of Gynecologic Surgery and Obstetrics, Uniformed Services University of the Health Sciences, Bethesda, MD (Dr Kuenstler and Dr Lutgendorf); 3 Department of Mathematics and Statistics, Kenyon College, Gambier, OH (Dr Snipes)

**Keywords:** obstetrical disparities, obstetrical inequities, racial disparities, racial inequities

## Abstract

**BACKGROUND:**

Racial and ethnic disparities in health care exist and are rooted in long-standing systemic inequities. These disparities result in significant excess health care expenditures and are due to complex interactions between patients, health care providers and systems, and social and environmental factors. In perinatal care, these inequities also exist, with Black patients being 3 to 4 times more likely to die of childbirth compared with White patients. Similar health care inequities may also exist in the Military Health System despite universal health care coverage, stable employment, and social programs that benefit military families.

**OBJECTIVE:**

This study aimed to evaluate racial disparities in obstetrical outcomes in the Military Health System.

**STUDY DESIGN:**

This is a retrospective cohort study of deliveries from 2019 to 2021 in the Military Health System, which provides obstetrical care for approximately 35,000 annual deliveries. The study was conducted using National Perinatal Information Center data on cesarean delivery, postpartum hemorrhage, and severe maternal morbidity by race and ethnicity from direct-care military hospitals representing tertiary care medical centers and community hospitals in the United States and abroad. Chi-square analyses and binary logistic regression were used to compare groups.

**RESULTS:**

The cohort included 68,918 deliveries. Of these, 32,358 (47%) were White, 9594 (13.9%) Black, 3120 (4.5%) Asian Pacific Islander, 456 (0.7%) American Indian/Alaska Native, 19,543 (28.4%) other, 3976 (5.8%) unknown, 7096 (10.3%) Hispanic, 58,009 (84.2%) non-Hispanic, and 4399 (6.4%) other ethnicity. Rates of cesarean delivery were significantly higher for Black (30%; odds ratio, 1.44; 95% confidence interval, 1.37–1.52), Asian Pacific Islander (27%; odds ratio, 1.24; 95% confidence interval, 1.14–1.35), and other (26%; odds ratio, 1.20; 95% confidence interval, 1.15–1.25) compared with White race (23%) (*P*<.001). Postpartum hemorrhage rates were higher for Black (5.9%; odds ratio, 1.11; 95% confidence interval, 1.00–1.24) and Asian Pacific Islander (7.7%; odds ratio, 1.49; 95% confidence interval, 1.29–1.72) compared with White race (5.3%) (*P*<.001). Severe maternal morbidity was higher for Black (2.9%; odds ratio, 1.44; 95% confidence interval, 1.24–1.67), Asian Pacific Islander (2.9%; odds ratio, 1.45; 95% confidence interval, 1.15–1.82), and other (2.8%; odds ratio, 1.36; 95% confidence interval, 1.21–1.54) compared with White race (2.1%) (*P*<.001). For severe maternal morbidity excluding blood transfusions, rates were also significantly higher for Black (1%; odds ratio, 1.68; 95% confidence interval, 1.30–2.17) than for White race (0.6%) (*P*<.002). Hispanic ethnicity was associated with a lower rate of severe maternal morbidity excluding transfusions (0.5%; odds ratio, 0.68; 95% confidence interval, 0.48–0.98) compared with non-Hispanic ethnicity (0.7%) (*P*=.04).

**CONCLUSION:**

Racial disparities in obstetrical outcomes exist in the Military Health System despite universal health care coverage, with significantly higher rates of cesarean delivery and severe maternal morbidity in Black, Asian Pacific Islander, and other races compared with White race. These findings suggest that these disparities are likely related to other factors or social determinants of health rather than availability of health care and insurance coverage. Further work should include investigation into such social determinants of health to address their causes, including systemic and structural barriers.

**AJOG Global Reports at a Glance**

**Why was this study conducted?**

This study aimed to evaluate racial disparities in obstetrical outcomes in a universally insured group of patients.

**Key findings**

This study found that racial disparities exist despite universal health care coverage, with higher rates of cesarean delivery and severe maternal morbidity in Black, Asian Pacific Islander, and other races compared with White race.

**What does this add to what is known?**

This study emphasizes the importance of factors other than health insurance and access to care, such as social determinants of health and systemic and structural barriers to care.

## Introduction

Racial and ethnic disparities exist in health care, resulting in excess expenditures of $135 billion annually, including $93 billion in health care costs and $42 billion in lost productivity.^1^ Such inequities can be related to health insurance coverage, access to care, quality of care, socioeconomic factors, and factors such as stereotyping, unconscious bias, racism, and language barriers.^2^ Health and health care inequities also exist in pregnancy and perinatal care, with Black patients at 3 to 4 times greater risk of death in childbirth compared with White patients, and 2 to 3 times greater risk of significant morbidity compared with White patients.^3^, ^4^, ^5^ It has been estimated that if the incidence of severe maternal morbidity (SMM) in racial and ethnic minority patients was lowered to the rate of non-Hispanic White patients, the overall incidence of SMM would decrease by 15%.^5^ Black, Asian Pacific Islander (API), and Hispanic patients are also more likely to have cesarean deliveries (CDs) than their White counterparts.^6^ Studies have proposed that lack of access to health care resources, socioeconomic status, insurance, and differing incidences of chronic medical conditions may contribute to these disparities. However, the underlying cause of racial and ethnic inequities in pregnancy and childbirth remains unclear despite previous studies.

Patients within the US Military Health System (MHS) have unique circumstances that warrant study of potential disparities in health outcomes. The MHS offers the advantages of universal health care coverage through TRICARE at civilian health care facilities, access to care through military treatment facilities (MTFs), and the Defense Health Agency, which provides medical care for all patients regardless of socioeconomic status or location.^7^ Some studies have shown a benefit to MHS care, with higher colorectal cancer screening rates observed among Black patients in the MHS compared with those of the Black civilian population.^8^ Such equalizing forces in the MHS, which provides health care, insurance, and also controls and standards for quality of care, may mitigate some of the health care inequities that are commonly observed in civilian populations.

Patients within the MHS may expect equity of care regardless of race or ethnicity, and patient reports have demonstrated minimal to no difference in perception of access to and quality of care in the MHS between races and ethnicities.^9^ Unfortunately, pregnancy outcomes in the MHS may have persistent disparities given that a previous study demonstrated higher rates of CD, admission to the intensive care unit, and maternal morbidity among Black pregnant patients in the MHS compared with non-Hispanic White patients.^10^

The study objective expands on this previous study^10^ by including additional racial and ethnic categories that allow a more in-depth examination of potential racial and ethnic inequities in obstetrical outcomes within the MHS. The objective is to assess health care inequities in the MHS and identify if additional racial and ethnic disparities exist in a broader sample including more diverse races and ethnicities.

## Materials and Methods

This was an institutional review board–approved quality improvement study at Naval Medical Center San Diego, a large tertiary care facility. We conducted a retrospective cohort study using National Perinatal Information Center (NPIC) quality improvement data. NPIC is a multistate perinatal quality improvement database that represents over 685,000 perinatal discharges annually. The NPIC database comprises coded, deidentified, aggregate data and includes maternal delivery distribution by race and ethnicity. Information was abstracted from 40 direct-care MTFs that provided obstetrical care over a 2-year period from April 1, 2019 to March 31, 2021. Data from all facilities were aggregated for this study. Facilities represented included tertiary care medical centers and community hospitals in the United States and abroad in hospitals run by the Defense Health Agency. Patients included in the study were active-duty service members and their spouses or children eligible for care at an MTF.

Study outcomes included the aggregate rates of CD, postpartum hemorrhage (PPH), SMM, SMM excluding transfusions, and SMM among deliveries affected by hemorrhage (Table 1). SMM definitions were those published by the Centers for Disease Control and Prevention (CDC) and based on International Classification of Diseases, 10th Revision (ICD-10) codes. SMM was defined on the basis of 21 indicators outlined by the CDC (Table 2). Self-identified and reported races in the NPIC data included White, Black, API, American Indian/Alaska Native (AI/AN), other, and unknown. Chi-square analyses were used to compare groups. Statistical analysis was completed using Minitab software (Mintab LLC, State College, PA).

**Table 1:** Definitions of primary outcomes included in the study

Cesarean delivery among all deliveries	Delivery through abdominal and uterine incision
Postpartum hemorrhage among all deliveries	Estimated blood loss >1000 mL after delivery
Severe maternal morbidity among all deliveries	Percentage of deliveries with ≥1 of the 21 CDC-identified severe maternal morbidity indicators
Severe maternal morbidity excluding blood transfusions among all deliveries	Percentage of deliveries with ≥1 of the 21 CDC-identified severe maternal morbidity indicators excluding cases with blood transfusion coded as the only severe morbidity
Severe maternal morbidity among preeclampsia cases	Percentage of delivered preeclampsia patients with ≥1 of the 21 CDC-identified maternal morbidity indicators
Severe maternal morbidity among preeclampsia cases excluding blood transfusions	Percentage of delivered preeclampsia patients with ≥1 of the 21 CDC-identified maternal morbidity indicators excluding cases with blood transfusion coded as the only severe morbidity
Severe maternal morbidity among hemorrhage cases	Percentage of delivered hemorrhage patients with ≥1 of the 21 CDC-identified maternal morbidity indicators
Severe maternal morbidity among hemorrhage cases excluding blood transfusions	Percentage of delivered hemorrhage patients with ≥1 of the 21 CDC-identified maternal morbidity indicators excluding cases with blood transfusion coded as the only severe morbidity

*CDC*, Centers for Disease Control and Prevention.

**Table 2:** Severe Maternal Morbidity Indicators (as defined by the CDC) included in the study

1. Acute myocardial infarction
2. Aneurysm
3. Acute renal failure
4. Adult respiratory distress syndrome
5. Amniotic fluid embolism
6. Cardiac arrest/ventricular fibrillation
7. Conversion of cardiac rhythm
8. Disseminated intravascular coagulation
9. Eclampsia
10. Heart failure/arrest during surgery or procedure
11. Puerperal cerebrovascular disorders
12. Pulmonary edema/acute heart failure
13. Severe anesthesia complications
14. Sepsis
15. Shock
16. Sickle cell disease with crisis
17. Air and thrombotic embolism
18. Blood products transfusion
19. Hysterectomy
20. Temporary tracheostomy
21. Ventilation

## Results

From April 1, 2019 to March 31, 2021, data were collected on 68,916 deliveries. The racial distribution of patients was 32,358 (47%) White, 9594 (13.9%) Black, 3120 (4.5%) API, 456 (0.7%) AI/AN, 19,543 (28.4%) other, 3976 (5.8%) unknown race, 7096 (10.3%) Hispanic ethnicity, 58,009 (84.2%) non-Hispanic ethnicity, and 4399 (6.4%) other ethnicity. Table 3 shows outcomes stratified by race and compared with the NPIC database average and the MHS average.

**Table 3:** Selected outcomes by race and ethnicity

Race and ethnicity	Cesarean deliveries among all deliveries	Postpartum hemorrhages among all deliveries	SMM among all deliveries	SMM excluding blood transfusions among all deliveries	SMM among hemorrhage deliveries
NPIC database average	33.5%	4.8%	2.2%	0.9%	24.2%
MHS average	25.3%	5.4%	2.5%	0.7%	31.8%
White	23% (n=6824)	5.3% (n=1584)	2.1% (n=610)	0.6% (n=183)	29.6% (n=520)
Black	30% (n=2548) OR 1.44 95% CI 1.37–1.53	5.9% (n=494) OR 1.11 95% CI 1.00–1.24	2.9% (n=248) OR 1.44 95% CI 1.24–1.67	1.0% (n=87) OR 1.68 95% CI 1.30–2.17	31.6% (n=199) OR 1.1 95% CI 0.90–1.34
API	27% (n=774) OR 1.24 95% CI 1.14–1.25	7.7% (n=223) OR 1.49 95% CI 1.29–1.72	2.9% (n=85) OR 1.45 95% CI 1.15–1.82	0.9% (n=25) OR 1.41 95% CI 0.93–2.14	30.7% (n=78) OR 1.05 95% CI 0.79–1.4
AI/AN	26% (n=53) OR 1.16 95% CI 0.85–2.59	3.4% (n=7) OR 0.62 95% CI 0.29–1.32	1.9% (n=4) OR 0.95 95% CI 0.35–2.55	0.5% (n=1) OR 0.79 95% CI 0.11–5.65	27.3% (n=8) OR 0.89 95% CI 0.24–3.37
Other	26% (n=4532) OR 1.20 95% CI 1.15–1.25	5.4% (n=936) OR 1.01 95% CI 0.94–1.11	2.8% (n=481) OR 1.36 95% CI 1.21–1.54	0.7% (n=121) OR 1.14 95% CI 0.90–1.43	35.8% (n=776) OR 1.33 95% CI 1.13–1.55
	*P*<.001	*P*<.001	*P*<.001	*P*=.002	*P*=.011
Non-Hispanic	26% (n=13,221)	5.3% (n=2752)	2.6% (n=2850)	0.7% (n=388)	31.3% (n=1091)
Hispanic	25% (n=624) OR 0.98 95% CI 0.92–1.04	5.6% (n=353) OR 1.06 95% CI 0.95–1.19	2.6% (n=162) OR 1.00 95% CI 0.84–1.18	0.5% (n=33) OR 0.68 95% CI 0.48–0.98	34.9% (n=145) OR 1.18 95% CI 0.95–1.45
	*P*=.42	*P*=.29	*P*=.97	*P*=.04	*P*=.13

Chi-square analysis by race (White, Black, API, AI/AN, and other) and ethnicity (Hispanic and non-Hispanic).

*AI/AN*, American Indian/Alaska Native; *API*, Asian Pacific Islander; *CI*, confidence interval; *MHS*, Military Health System; *NPIC*, National Perinatal Information Center; *OR*, odds ratio; *SMM*, severe maternal morbidity.

**Figure 1:**
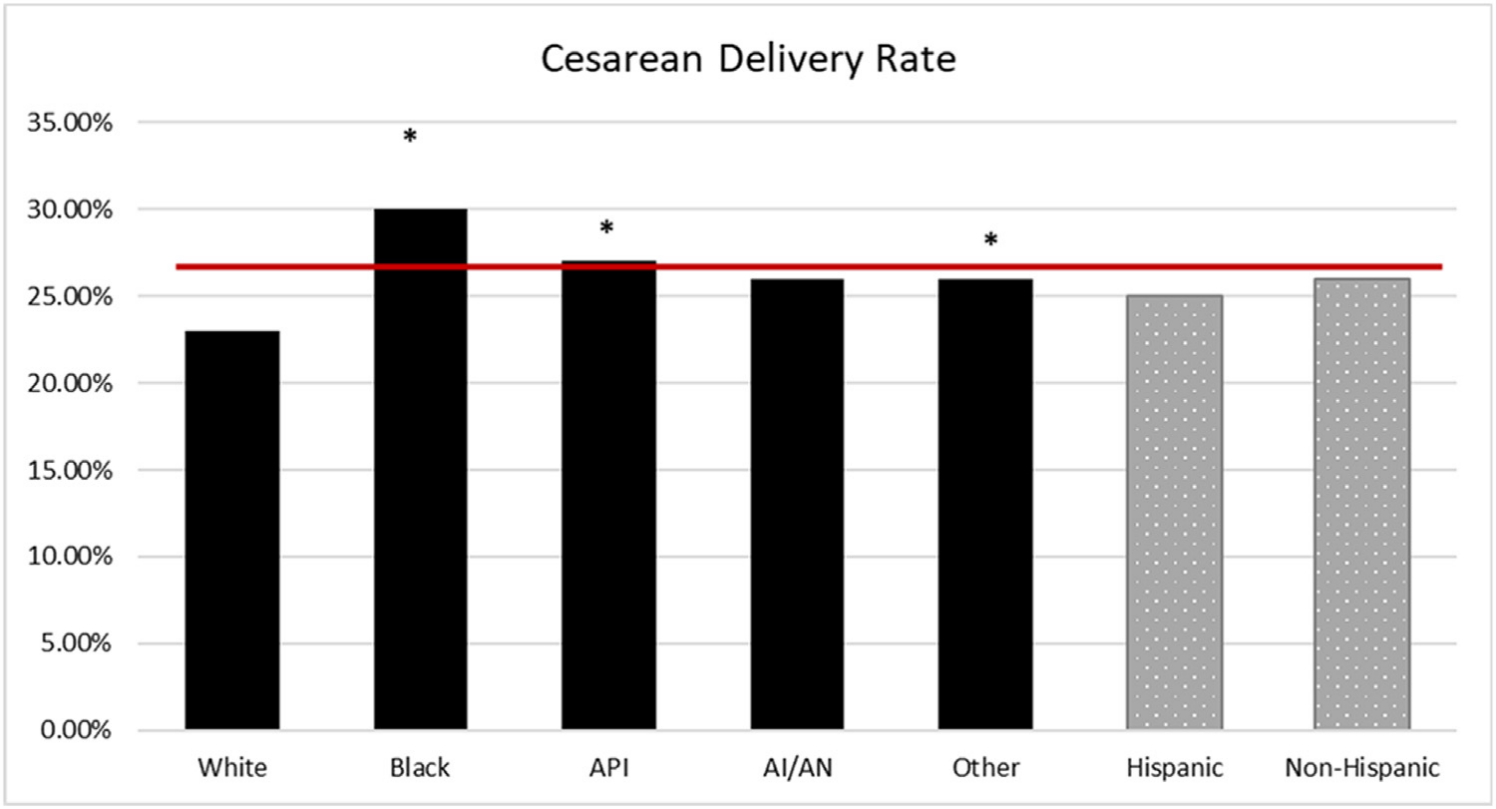
Rate of cesarean delivery stratified by race and ethnicity The (*asterisk*) represents significant differences across race and ethnicity categories using chi-square testing. The solid line represents the Military Health System average rate. *AI/AN*, American Indian/Alaska Native; *API*, Asian Pacific Islander.

**Figure 2:**
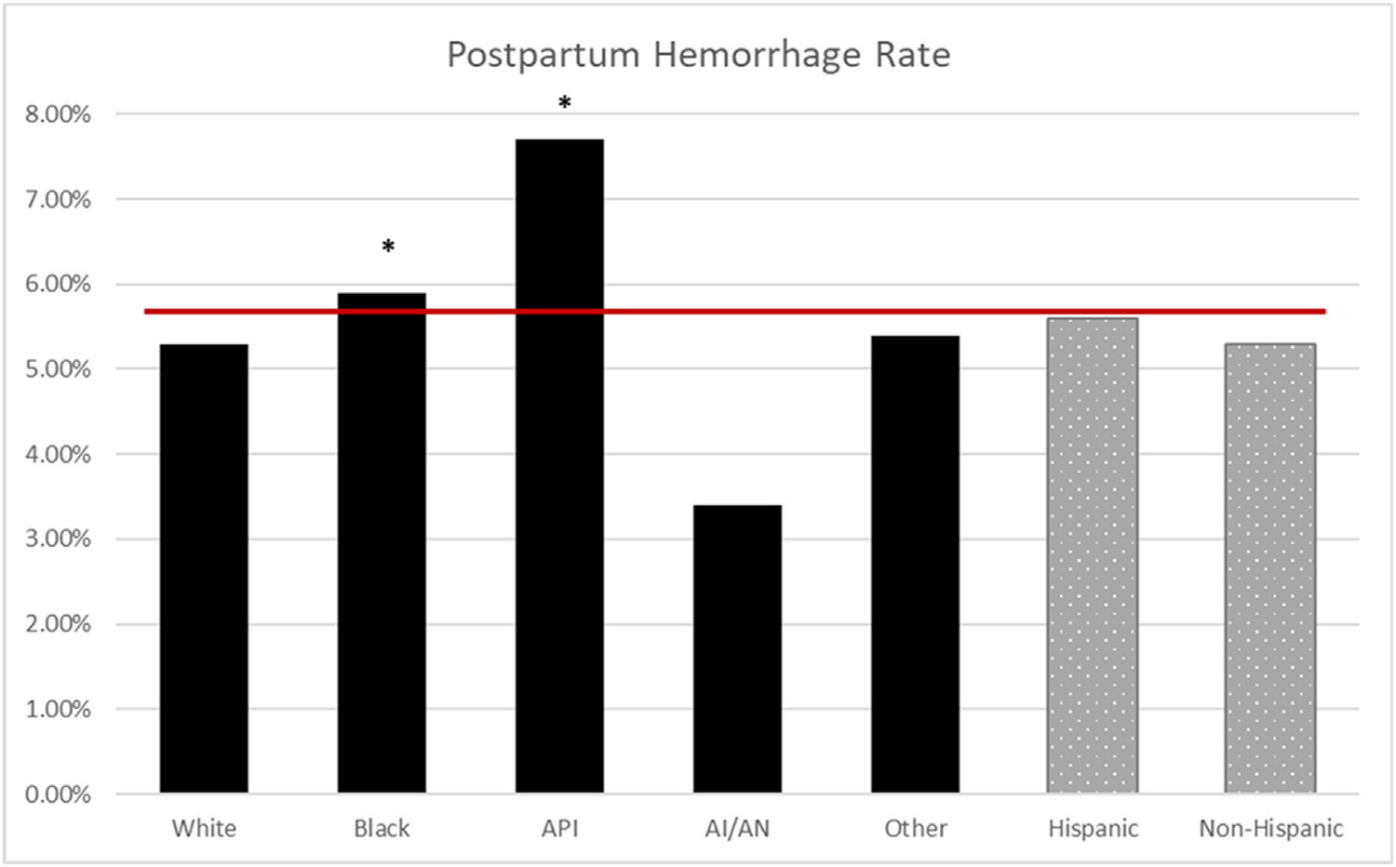
Rate of postpartum hemorrhage stratified by race and ethnicity The (*asterisk*) represents significant differences in odds ratios across race and ethnicity categories. The solid line represents the Military Health System average rate. *AI/AN*, American Indian/Alaska Native; *API*, Asian Pacific Islander.

**Figure 3:**
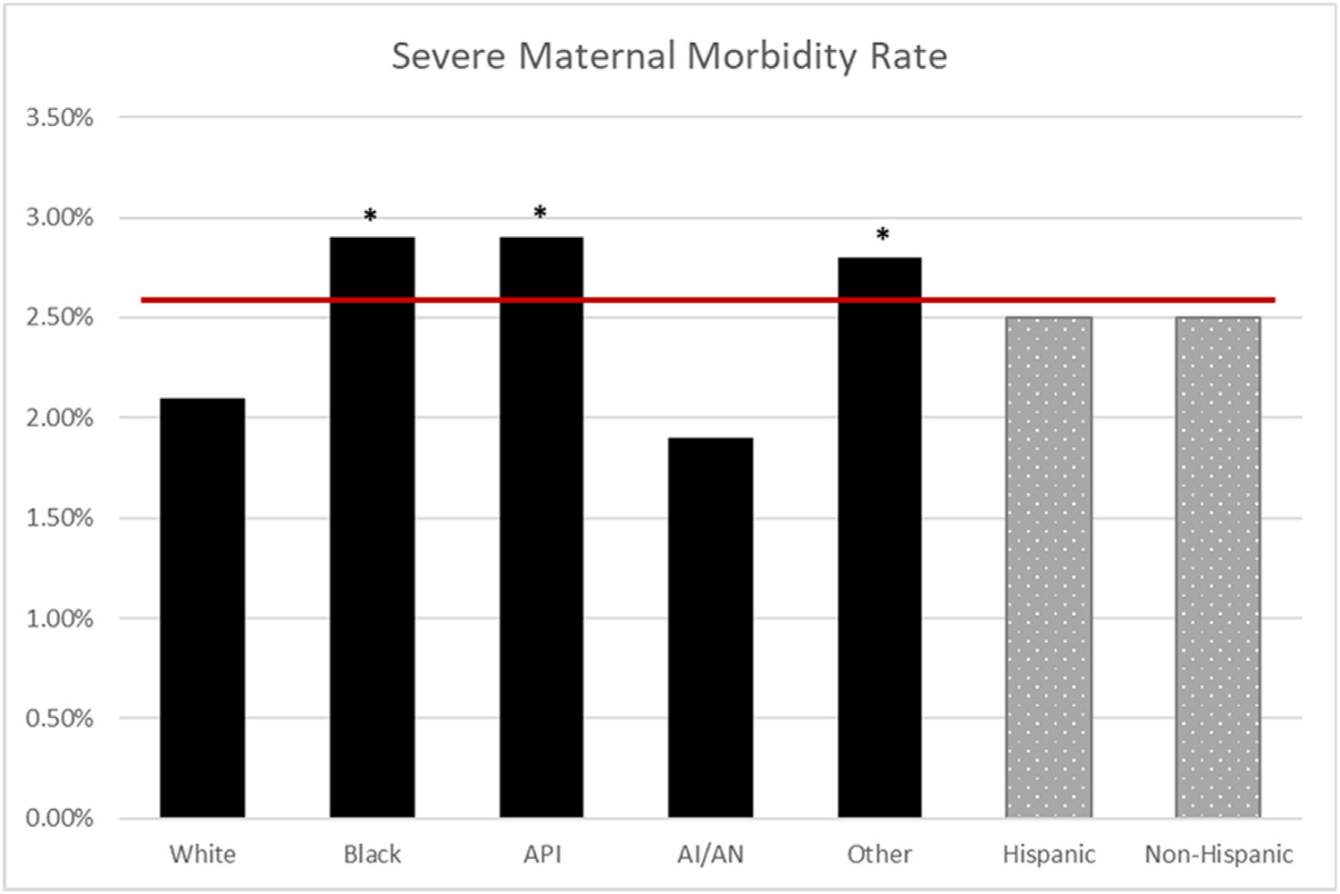
Rate of severe maternal morbidity stratified by race and ethnicity The (*asterisk*) represents significant differences in odds ratios across race and ethnicity categories. The solid line represents the Military Health System average rate. *AI/AN*, American Indian/Alaska Native; *API*, Asian Pacific Islander.

**Figure 4:**
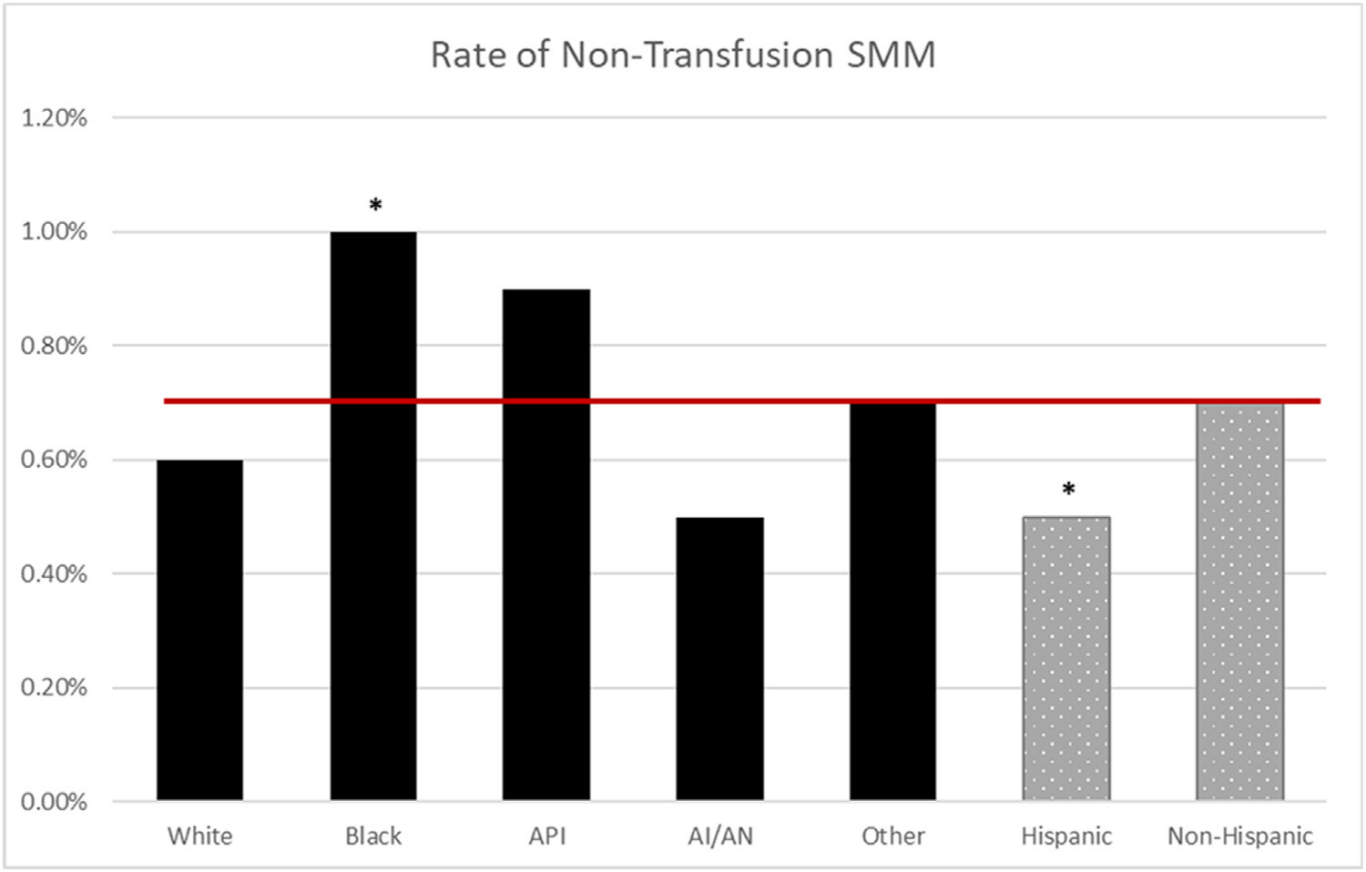
Rate of SMM excluding transfusion stratified by race The (*asterisk*) represents significant differences in odds ratios across race and ethnicity categories. The solid line represents the Military Health System average rate. *AI/AN*, American Indian/Alaska Native; *API*, Asian Pacific Islander; *SMM*, severe maternal morbidity.

**Figure 5:**
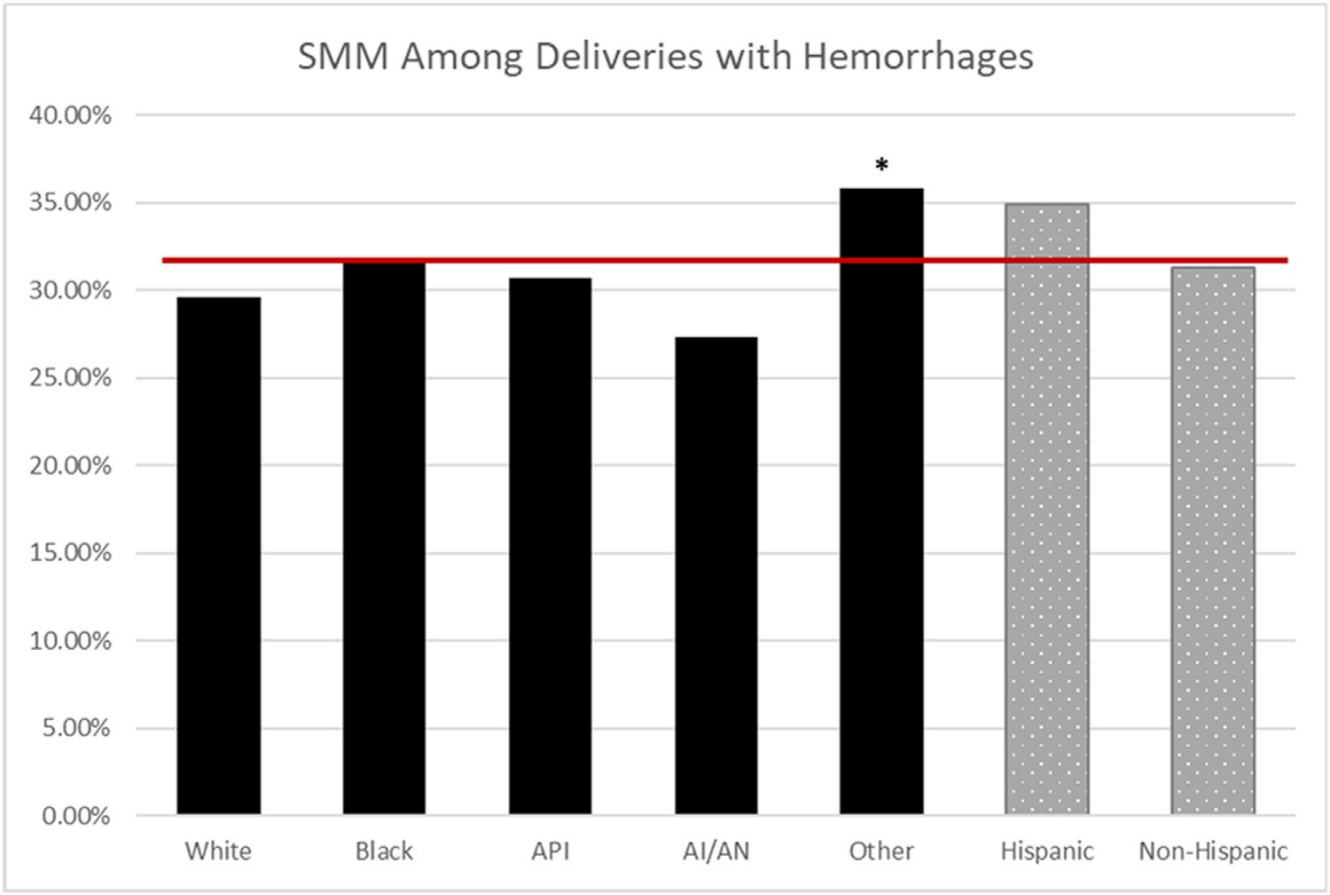
Rate of SMM among hemorrhage deliveries stratified by race The (*asterisk*) represents significant differences in odds ratios across race and ethnicity categories. The solid line represents the Military Health System average rate. *AI/AN*, American Indian/Alaska Native; *API*, Asian Pacific Islander; *SMM*, severe maternal morbidity.

The NPIC average for CD in the MHS was 25.3%; CD rates were higher for Black (30%; odds ratio [OR], 1.44; 95% confidence interval [CI], 1.37–1.52), API (27%; OR, 1.24; 95% CI, 1.14–1.35), and other race (26%; OR, 1.20; 95% CI, 1.15–1.25) compared with White race (23%) (*P*<.001) (Figure 1). The NPIC average for PPH in the MHS was 5.4%, and PPH rates were higher for Black (5.9%; OR, 1.11; 95% CI, 1.00–1.24) and API (7.7%; OR, 1.49; 95% CI, 1.29–1.72) compared with White race (5.3%) (*P*<.001) (Figure 2). The NPIC average for SMM in the MHS was 2.5%, and SMM rates were higher for Black (2.9%; OR, 1.44; 95% CI, 1.24–1.67), API (2.9%; OR, 1.45; 95% CI, 1.15–1.82), and other race (2.8%; OR, 1.36; 95% CI, 1.21–1.54) compared with White race (2.1%) (*P*<.001) (Figure 3). The NPIC average for SMM excluding transfusions in the MHS was 0.7%, and rates of SMM excluding blood transfusions were significantly higher for Black (1%; OR, 1.67; 95% CI, 1.30–2.17) than for White race (0.6%) (*P*=.002), and significantly lower for Hispanic (0.5%; OR, 0.68; 95% CI, 0.48–0.98) than for non-Hispanic ethnicity (0.7%) (*P*=.04) (Figure 4). The NPIC average for SMM among deliveries complicated by hemorrhage was 31.8%, and rates of SMM among hemorrhage deliveries were also higher for other race (35.8%; OR, 1.33; 95% CI, 1.13–1.55) compared with White race (29.6%) (*P*=.011) (Figure 5).

Fuller. Obstetrical health care disparities. Am J Obstet Gynecol Glob Rep 2023.

Fuller. Obstetrical health care disparities. Am J Obstet Gynecol Glob Rep 2023.

Fuller. Obstetrical health care disparities. Am J Obstet Gynecol Glob Rep 2023.

Fuller. Obstetrical health care disparities. Am J Obstet Gynecol Glob Rep 2023.

Fuller. Obstetrical health care disparities. Am J Obstet Gynecol Glob Rep 2023.

When stratifying patients by race, Black patients had the highest CD rate of 30% and API patients the highest PPH rate of 7.7%. Black, API and other race had the highest rates of SMM at 2.8% to 2.9%. Black patients also had the highest rates of SMM after excluding transfusions at 1%, and other race had the highest rates of SMM among deliveries affected by hemorrhage.

## Discussion

### Principal findings

These findings demonstrate significant racial and ethnic inequities in obstetrical outcomes in the MHS despite universal health care coverage and access to care. Significant disparities were found among non-White races for CD, PPH, and SMM.

### Results

Besides the previously reported disparities for Black women,^10^ the data demonstrated additional racial inequities in the MHS. This study found inequities in rates of CD, PPH, and SMM for patients who identified as Black, API, other race, and Hispanic. These findings are similar to disparities reported in civilian obstetrical populations. Although significant inequities were not found for AI/AN, this group also had a relatively low number of patients (0.7% of the population). Thus, it does not appear that universal health care coverage and access to care in the MHS eliminate health care inequities during the delivery hospitalization.

Previous studies in the civilian setting have noted that such inequities persisted even when data were adjusted for patient risk factors.^5^ Grobman et al^11^ analyzed a nationwide cohort of patients over a 3-year period. After adjusting for patient characteristics and comorbid conditions such as diabetes mellitus and hypertension, they found that significant disparities persisted in rate of PPH, peripartum infection, severe perineal laceration, and a number of other related outcomes.^11^ Because we did not control for comorbidities of individual patients, we are unable to determine whether some of these differences could be due to differences in underlying comorbidities.

### Clinical implications

Because health care disparities persist despite insurance coverage and access to care, clinicians and health care providers must work to address inequities in their practices, and advocate for systemic changes. Individual-level opportunities for providers include work to enhance trust, mutual respect, and understanding in their patient relationships.^2^ Such actions should include connecting with patients empathetically and developing a personal relationship that humanizes the patient and caregiver, which can ultimately reduce stereotypes and increase trust.^2^ Other strategies include patient-centered education with teach-backs, using medical interpreters when needed, consideration of health literacy, welcoming the patient's family and support persons, and encouraging patients to ask questions and complete patient satisfaction and demographic forms.^2^ Being aware of social determinants of health is also important given that lower educational attainment and lower health literacy have been associated with less understanding of pregnancy complications such as diabetes mellitus and hypertensive disorders of pregnancy.^12^^,^^13^

The first step to effect systemic change is consistent and reliable measurement of outcomes to identify disparities and develop meaningful solutions.^14^ Use of databases such as NPIC for monitoring outcomes data with stratification by race and ethnicity allows ongoing monitoring and assessment of the effectiveness of interventions for decreasing racial disparities. Addressing implicit and institutional bias, and creating a just culture are effective interventions at a systems level that can decrease disparities.^14^ Further addressing the effects of social determinants of health and systemic racism on obstetrical health and health care disparities is another important area of clinical focus. Efforts may include internal assessments of barriers and facilitators of equitable care, data monitoring and assessment for disparities in outcomes, and screening for social determinants or health with effective referrals to social support services.^15^ In the MHS, such resources exist, including community-based resources and additional military services such as those provided by Military OneSource and the Military and Family Support Centers. Continued identification of patients who would benefit from additional support and timely referrals are important components of reducing health care inequities.

### Research implications

Previous research indicates that substantial health care disparities exist in perinatal health outcomes,^3^, ^4^, ^5^, ^6^^,^^10^^,^^11^^,^^15^^,^^16^ and that infrastructure changes are needed to address necessary changes in research paradigms and praxis while addressing structural racism.^16^ The results of this study support that health care inequities exist even in systems with universal health care coverage. Future studies should examine specific social determinants of health that may be underlying these differences to determine interventions that may improve outcomes and decrease inequities. Ongoing assessments of trends over time in racial and ethnic disparities in perinatal outcomes will allow for monitoring of the effectiveness of interventions. Health care systems should continue to adopt practices and monitor perinatal quality metrics stratified by race and ethnicity to reduce and eliminate inequities. Such quality and safety strategies include identifying the root causes of inequities, applying implementation science to improve systems and processes, and using data to further refine and improve the process.^17^

### Strengths and limitations

A strength of this study is the inclusion of data from a large health care system with a variety of practice settings, including medical centers and community hospital, urban, rural, and overseas settings. Thus, the study findings are generalizable across different practice settings and geographic locations. The use of the NPIC database is another strength because the database uses coded data that are validated before inclusion.

This study also has several limitations due to the use of the NPIC database. The database includes only aggregate deidentified data reported by the facility. Thus, we were unable to assess individual patient-level factors that may have contributed to obstetrical outcomes. We were also unable to assess factors that may have contributed to inequities in care delivery or access. Because of the nature of the NPIC data, we were also unable to assess differences in neonatal outcomes related to race and ethnicity. However, a recent publication on neonatal mortality in the MHS showed persistent racial disparities, with a 2-fold higher neonatal mortality rate for non-Hispanic Black neonates compared with White neonates despite an overall lower neonatal mortality rate in the MHS.^18^ This difference was likely related to an overall higher rate of preterm birth in non-Hispanic Black neonates in this cohort.^18^ Another limitation is that the NPIC database only includes individuals who actually received care in the MHS, and does not include patients who had coverage but might have been unable to obtain care in the system. Another limitation was that approximately one-third of the self-identified race data were classified as unknown (5%) or other (28%), and this proportion varied among facilities. This may be due to an increasing prevalence of mixed racial backgrounds in the military and missing race identification reporting in the Defense Enrollment Eligibility Reporting System (DEERS). Currently, race data collected in the DEERS system do not allow reporting for >1 race. Although a significant percentage of the data do not have a singular race identification, the “Other” race identification may still hold statistical value given that persons identifying as “Other” may phenotypically present as a person of non-White race, which has the potential to affect the care that they receive. We also found significantly higher CD, PPH, and SMM rates for patients who identified as other race.

Another limitation was the relatively small percentage of AI/AN patients receiving care, representing <1% of the population. Because of such a small sample size, the observed inequities for AI/AN patient populations were not statistically significant. Finally, the data were collected over a 2-year period that encompassed the COVID-19 pandemic, which may also have affected the outcomes.

### Conclusions

Racial disparities in obstetrical outcomes exist in the MHS despite universal health care coverage, and are likely related to other systemic factors or social determinants of health. Further studies are warranted to evaluate these factors and other social determinants of health that may be contributing to these inequities. An analysis of institutional policies should be performed to assess whether systemic racism or implicit bias is present at the institutional and provider levels. Impactful and introspective implicit bias training should be mandatory for all health care team members, with refresher courses biannually. Standardized pathways, such as for perinatal care, induction of labor, and PPH, should continue to be implemented, and are an integral part of providing equitable health care and decreasing nonbeneficial clinical variation.^19^
